# Supplementary material for: Recurrent mutations drive the rapid evolution of pesticide resistance in the two-spotted spider mite Tetranychus urticae
Source: eLife. 2025 Aug 11;14:RP106288. doi: 10.7554/eLife.106288 (PMC12339004; doi:10.7554/eLife.106288)
Supplement: Supplementary file 1. [file elife-106288-supp1.docx]

**Supplementary File 1. Population information of the two-spotted spider mite *Tetranychus* *urticae* used in this study**

| **Population** | **Date** | **Location** | **Host** | **Pool-seq** | **Sanger-seq** | **Bioassay** | **Data source** |
| --- | --- | --- | --- | --- | --- | --- | --- |
| BJCP1 | 2013.04.25 | Changping District, Beijing | strawberry |  | Y |  | This study |
| BJPG1 | 2013.05.23 | Pinggu District, Beijing | strawberry |  | Y |  | This study |
| BJHD1 | 2017.03.13 | Haidian District, Beijing | eggplant |  | Y |  | This study |
| SXYQ | 2017.03.14 | Yangquan City, Shanxi Province | strawberry | Y |  |  | This study |
| AHHN | 2017.03.16 | Huainan City, Anhui Province | strawberry | Y |  |  | This study |
| SDRZ | 2017.03.22 | Rizhao City, Shandong Province | strawberry | Y | Y |  | This study |
| SDSG1 | 2017.03.29 | Shouguang City, Shandong Province | pepper |  | Y |  | This study |
| HNHK | 2017.03.30 | Haikou City, Hainan Province | kidney beans | Y | Y |  | This study |
| SCCD1 | 2017.03.30 | Chengdu City, Sichuan Province | strawberry | Y | Y |  | This study |
| SHPD | 2017.04.13 | Pudong District, Shanghai | strawberry | Y | Y |  | This study |
| HNCS1 | 2017.04.02 | Changsha City, Hunan Province | strawberry | Y | Y |  | This study |
| BJTZ1 | 2017.04.24 | Tongzhou District, Beijing | strawberry |  | Y |  | This study |
| BJPG2 | 2017.05.05 | Pinggu District, Beijing | strawberry |  | Y |  | This study |
| JXNC | 2017.05.08 | Nanchang City, Jiangxi Province | strawberry | Y |  |  | This study |
| BJCP2 | 2017.07.26 | Changping District, Beijing | strawberry |  | Y |  | This study |
| BJDX | 2018 | Daxing District, Beijing | strawberry |  |  | Y | Gong et al. 2018 |
| BJDX1 | 2018.02 | Daxing District, Beijing | strawberry |  |  | Y | Chen et al. 2019 |
| BJDX2 | 2018.04 | Daxing District, Beijing | strawberry |  |  | Y | Chen et al. 2019 |
| BJFS | 2018.04 | Fangshan District, Beijing | strawberry |  |  | Y | Chen et al. 2019 |
| ZJXS1 | 2018.04 | Hangzhou City, Zhejiang Province | strawberry |  | Y | Y | Chen et al. 2019 |
| BJSY | 2018.05 | Shunyi District, Beijing | strawberry |  |  | Y | Chen et al. 2019 |
| ZJWX | 2018.05 | Wuxing City, Zhejiang province | strawberry |  |  | Y | Chen et al. 2019 |
| ZJHZ | 2020.04.14 | Hangzhou City, Zhejiang Province | strawberry |  |  | Y | This study |
| HBBD | 2020.04.15 | Baoding City, Hebei Province | strawberry |  |  | Y | This study |
| HNZZ | 2020.04.15 | Zhengzhou City, Henan Province | strawberry |  | Y | Y | This study |
| SDSG2 | 2020.04.16 | Shouguang City, Shandong Province | kidney beans |  | Y | Y | This study |
| BJDX3 | 2020.04.17 | Daxing District, Beijing | strawberry |  |  | Y | This study |
| BJYQ | 2020.04.17 | Yanqing District, Beijing | strawberry |  |  | Y | This study |
| SXAK | 2020.04.17 | Ankang City, Shaanxi Province | strawberry |  | Y | Y | This study |
| ZJJX | 2020.04.17 | Jiaxing City, Zhejiang Province | strawberry |  |  | Y | This study |
| ZJNB | 2020.04.17 | Ningbo City, Zhejiang Province | strawberry |  |  | Y | This study |
| NMHH1 | 2020.04.18 | Hohhot City, Inner Mongolia Autonomous Region | strawberry |  |  | Y | This study |
| SDQD | 2020.04.20 | Qingdao City, Shandong Province | strawberry |  | Y | Y | This study |
| YNKM1 | 2020.04.23 | Kunming City, Yunnan Province | rose |  | Y | Y | This study |
| BJHD2 | 2020.04.26 | Haidian District, Beijing | strawberry |  | Y | Y | This study |
| LNSY | 2020.05.22 | Shenyang City, Liaoning Province | strawberry |  | Y | Y | This study |
| YNKM2 | 2021.11.03 | Kunming City, Yunnan Province | rose |  | Y | Y | This study |
| BJCP4 | 2021.04.14 | Changping District, Beijing | strawberry | Y | Y | Y | This study |
| SDSG3 | 2021.04.18 | Shouguang City, Shandong Province | cucumber | Y | Y | Y | This study |
| SDSG4 | 2021.04.18 | Shouguang City, Shandong Province | eggplant |  | Y | Y | This study |
| BJCP5 | 2021.04.27 | Changping District, Beijing | strawberry |  |  | Y | This study |
| BJPG3 | 2021.04.27 | Pinggu District, Beijing | strawberry |  | Y |  | This study |
| SCCD2 | 2021.06.12 | Chengdu City, Sichuan Province | eggplant |  | Y |  | This study |
| NMHH2 | 2021.06.13 | Hohhot City, Inner Mongolia Autonomous Region | kidney beans | Y | Y | Y | This study |
| SDQZ | 2021.06.13 | Qingzhou City, Shandong Province | eggplant | Y | Y | Y | This study |
| NXGY2 | 2021.06.27 | Guyuan City, Ningxia Province | cucumber |  | Y | Y | This study |
| BJHD3 | 2021.07.23 | Haidian District, Beijing | corn |  | Y |  | This study |
| SDSG5 | 2021.07.07 | Shouguang City, Shandong Province | eggplant |  | Y | Y | This study |
| SDSG6 | 2021.07.07 | Shouguang City, Shandong Province | pepper |  | Y | Y | This study |
| BJTZ2 | 2021.04.30 | Tongzhou District, Beijing | strawberry |  | Y |  | This study |
| BJMY | 2024.01.30 | Miyun District, Beijing | strawberry |  |  | Y | This study |
| SDSG7 | 2024.01.30 | Shouguang City, Shandong Province | cucumber |  |  | Y | This study |
| BJHD4 | 2024.02.24 | Haidian District, Beijing | strawberry |  | Y | Y | This study |
| BJDX5 | 2024.03.15 | Daxing District, Beijing | strawberry |  | Y | Y | This study |
| BJDX6 | 2024.03.15 | Daxing District, Beijing | strawberry | Y | Y | Y | This study |
| BJDX7 | 2024.01.30 | Daxing District, Beijing | strawberry |  | Y | Y | This study |
| GXNN | 2024.03.31 | Naning City, Guangxi Province | strawberry | Y | Y | Y | This study |
| GZGY | 2024.04.03 | Guiyang City, Guizhou Province | strawberry |  | Y |  | This study |
| HNCS2 | 2024.01.29 | Changsha City, Hunan Province | strawberry | Y | Y | Y | This study |
| JXYC | 2024.03.10 | Yichun City, Jiangxi Province | strawberry |  | Y |  | This study |
| LNDD | 2024.03.10 | Dandong City, Liaoning Province | strawberry | Y | Y | Y | This study |
| QHHD | 2024.02.29 | Haidong City, Qinghai Province | strawberry | Y | Y | Y | This study |
| SCCD3 | 2024.04.08 | Chengdu City, Sichuan Province | strawberry |  | Y |  | This study |
| SDSG8 | 2024.03.12 | Shouguang City, Shandong Province | strawberry |  | Y |  | This study |
| SDWF | 2024.04.10 | Weifang City, Shandong Province | eggplant |  | Y |  | This study |
| YNKM3 | 2024.04.07 | Kunming City, Yunnan Province | strawberry |  | Y |  | This study |
| YNKM4 | 2024.04.04 | Kunming City, Yunnan Province | pepper |  | Y |  | This study |
| YNYX | 2024.03.5 | Yuxi City, Yunnan Province | strawberry | Y | Y | Y | This study |
| ZJHZ2 | 2024.03.15 | Hangzhou City, Zhejiang Province | strawberry | Y | Y | Y | This study |
| ZJHZ3 | 2024.03.15 | Hangzhou City, Zhejiang Province | strawberry | Y | Y | Y | This study |
| LabS | 2018.04 | Hangzhou City, Zhejiang Province | strawberry | Y |  | Y | This study |
| LabR | 2018.04 | Hangzhou City, Zhejiang Province | strawberry | Y | Y | Y | This study |

Y.-J. Gong et al., Toxicity and field control efficacy of the new acaricide SYP-9625 to the two-spotted spider mite (Tetranychus urticae koch). Agrochemicals 56, 561-563 (2017).

J. C. Chen et al., Field-evolved resistance and cross-resistance of the two-spotted spider mite, Tetranychus urticae, to bifenazate, cyenopyrafen and SYP-9625. Experimental and Applied Acarology (2.38) 77, 545-554 (2019).
