# Supplementary material for: Recurrent mutations drive the rapid evolution of pesticide resistance in the two-spotted spider mite Tetranychus urticae
Source: eLife. 2025 Aug 11;14:RP106288. doi: 10.7554/eLife.106288 (PMC12339004; doi:10.7554/eLife.106288)
Supplement: Supplementary file 2. [file elife-106288-supp2.docx]

**Supplementary File 2. Mutant allele frequency (Sanger / pooled sequencing) and susceptibility of the two-spotted spider mites *Tetranychus* *urticae* to cyetpyrafen.** #, from Gong et al. 2018; $, from Chen et al., 2019. Sample size (sdhB/sdhD). Frequency of mutant individuals (carrying any of the mutation) is calculated from Sanger sequencing data of all mutations except for H146Q, S212I, A285S, D116E, and R119P mutations. SR, survival rate (%) under 1000 mg/L. LC_50_ (mg/L), number of proportions shows mortality treated by 32000 mg/L.

| **Population** | **Year** | ***B*_H146Q** | ***B_*S212I** | ***B_H258Y*** | ***B_*I260T** | ***B_*I260V** | ***B_*A285S** | ***D_*D116G** | ***D_*D116E** | ***D_*D116N** | ***D_*R119C** | ***D_*R119G** | ***D_*R119L** | ***D_*R119P** | ***D_*R119H** | ***D_P120L*** | **No. mutation** | **Mutant indiv. %** | **LC_50_ (mg/L)** | **SR** |
| --- | --- | --- | --- | --- | --- | --- | --- | --- | --- | --- | --- | --- | --- | --- | --- | --- | --- | --- | --- | --- |
| BJCP1 | 2013 | - | - | 0/- | 0/- | 0/- | 0/- | 0/- | 0/- | 0/- | 0/- | 0/- | 0/- | 0/- | 0/- | 0/- | 0 | 0 | - | - |
| BJPG1 | 2013 | - | - | 0/- | 0/- | 0/- | 0/- | 0/- | 0/- | 0/- | 0/- | 0/- | 0/- | 0/- | 0/- | 0/- | 0 | 0 | - | - |
| BJCP2 | 2017 | - | - | 0/- | 0/- | 0/- | 0/- | 0/- | 0/- | 0/- | 0/- | 0/- | 0/- | 0/- | 0/- | 0/- | 0 | 0 | - | - |
| BJHD1 | 2017 | - | - | 0/- | 0/- | 0/- | 0/- | 0/- | 0/- | 0/- | 0/- | 0/- | 0/- | 0/- | 0/- | 0/- | 0 | 0 | - | - |
| BJPG2 | 2017 | - | - | 0/- | 0/- | 0/- | 0/- | 0/- | 0/- | 0/- | 0/- | 0/- | 0/- | 0/- | 0/- | 0/- | 0 | 0 | - | - |
| SXYQ | 2017 | -/0 | -/0 | -/0 | -/0 | -/0 | -/0 | -/0 | -/0 | -/0 | -/0 | -/0 | -/0 | -/0 | -/0 | -/0 | 0 | - | - | - |
| AHHN | 2017 | -/0 | -/0 | -/0 | -/0 | -/0 | -/0 | -/0 | -/0 | -/0 | -/0 | -/0 | -/0 | -/0 | -/0 | -/0 | 0 | - | - | - |
| JXNC | 2017 | -/0 | -/0 | -/0 | -/0 | -/0 | -/0 | -/0 | -/0 | -/0 | -/0 | -/0 | -/0 | -/0 | -/0 | -/0 | 0 | - | - | - |
| BJTZ1 | 2017 | - | - | 0/- | 0/- | 0/- | 0/- | 0/- | 0/- | 0/- | 0/- | 0/- | 0/- | 0/- | 0/- | 0/- | 0 | 0 | - | - |
| HNCS1 | 2017 | -/0 | -/0 | 0/0 | 0/0 | 0/0 | 0/0 | 0/0 | 0/0 | 0/0 | 0/0 | 0/0 | 0/0 | 0/0 | 0/0 | 0/0 | 0 | 0 | - | - |
| HNHK | 2017 | -/0 | -/0 | 0/0 | 0/0 | 0/0 | 0/0 | 0/0 | 0/0 | 0/0 | 0/0 | 0/0 | 0/0 | 0/0 | 0/0 | 0/0 | 0 | 0 | - | - |
| SCCD1 | 2017 | -/0 | -/0 | 0/0 | 0/0 | 0/0 | 0/0 | 0/0 | 0/0 | 0/0 | 0/0 | 0/0 | 0/0 | 0/0 | 0/0 | 0/0 | 0 | 0 | - | - |
| SDRZ | 2017 | -/0 | -/0 | 0/0 | 0/0 | 0/0 | 1.9/33 | 0/0 | 0/0 | 0/0 | 0/0 | 0/0 | 0/0 | 0/0 | 0/0 | 0/0 | 1 | 0 | - | - |
| SDSG1 | 2017 | - | - | 0/- | 0/- | 0/- | 0/- | 0/- | 0/- | 0/- | 0/- | 0/- | 0/- | 0/- | 0/- | 0/- | 0 | 0 | - | - |
| SHPD | 2017 | -/0 | -/0 | 0/0 | 0/0 | 0/0 | 0/0 | 0/0 | 0/0 | 0/0 | 0/0 | 0/0 | 0/0 | 0/0 | 0/0 | 0/0 | 0 | 0 | - | - |
| BJDX# | 2018 | - | - | - | - | - | - | - | - | - | - | - | - | - | - | - | - | - | 9.64 | - |
| BJDX1$ | 2018 | - | - | - | - | - | - | - | - | - | - | - | - | - | - | - | - | - | 2.51 | - |
| BJDX2$ | 2018 | - | - | - | - | - | - | - | - | - | - | - | - | - | - | - | - | - | 2.15 | - |
| BJFS$ | 2018 | - | - | - | - | - | - | - | - | - | - | - | - | - | - | - | - | - | 4.15 | - |
| ZJXS1$ | 2018 | - | - | 0/- | 0/- | 0/- | 0/- | 0/- | 0/- | 0/- | 0/- | 0/- | 0/- | 0/- | 0/- | 0/- | 0 | 0 | 21.55 | 0 |
| BJSY$ | 2018 | - | - | - | - | - | - | - | - | - | - | - | - | - | - | - | - | - | 1.4 | - |
| ZJWX$ | 2018 | - | - | - | - | - | - | - | - | - | - | - | - | - | - | - | - | - | 6.54 | - |
| HNZZ | 2020 | - | - | 0/- | 0/- | 0/- | 0/- | 0/- | 0/- | 0/- | 0/- | 0/- | 0/- | 0/- | 1.79/- | 0/- | 1 | 0 | 1.31 | 0 |
| BJDX3 | 2020 | - | - | - | - | - | - | - | - | - | - | - | - | - | - | - | - | - | 1.89 | - |
| BJHD2 | 2020 | - | - | 0/- | 0/- | 0/- | 0/- | 0/- | 0/- | 0/- | 0/- | 0/- | 0/- | 85.71/- | 0/- | 0/- | 1 | 0 | 12.95 | 0 |
| BJYQ | 2020 | - | - | - | - | - | - | - | - | - | - | - | - | - | - | - | - | - | 1.78 | - |
| HBBD | 2020 | - | - | - | - | - | - | - | - | - | - | - | - | - | - | - | - | - | 2.8 | - |
| LNSY | 2020 | - | - | 0/- | 0/- | 0/- | 0/- | 0/- | 0/- | 0/- | 25.0/- | 0/- | 3.57/- | 0/- | 0/- | 0/- | 2 | 27.27 | 4.04 | 0 |
| NMHH1 | 2020 | - | - | - | - | - | - | - | - | - | - | - | - | - | - | - | - | - | 1.72 | - |
| SDQD | 2020 | - | - | 0/- | 0/- | 0/- | 0/- | 0/- | 0/- | 3.45/- | 0/- | 0/- | 0/- | 0/- | 0/- | 0/- | 0 | 3.45 | 2.11 | 0 |
| SDSG2 | 2020 | - | - | 0/- | 12.5/- | 0/- | 0/- | 66.7/- | 0/- | 0/- | 0/- | 0/- | 0/- | 0/- | 0/- | 0/- | 2 | 100 | 14.7% | 85.7 |
| SXAK | 2020 | - | - | 0/- | 0/- | 0/- | 0/- | 0/- | 0/- | 0/- | 0/- | 0/- | 0/- | 0/- | 0/- | 0/- | 0 | 0 | 2.29 | 0 |
| YNKM1 | 2020 | - | - | 0/- | 3.8/- | 0/- | 0/- | 0/- | 0/- | 0/- | 0/- | 0/- | 0/- | 0/- | 0/- | 0/- | 1 | 5.56 | 7.57 | 0 |
| ZJHZ | 2020 | - | - | - | - | - | - | - | - | - | - | - | - | - | - | - | - | - | 4.27 | - |
| ZJJX | 2020 | - | - | - | - | - | - | - | - | - | - | - | - | - | - | - | - | - | 1.72 | - |
| ZJNB | 2020 | - | - | - | - | - | - | - | - | - | - | - | - | - | - | - | - | - | 1.97 | - |
| BJCP4 | 2021 | -/2.13 | -/0 | 0/0 | 1.9/0 | 44.2/54.5 | 0/0 | 0/0 | 0/0 | 0/0 | 78.1/82.8 | 0/0 | 0/0 | 0/0 | 0/0 | 0/0 | 4 | 100 | 13546.15 | 74.6 |
| BJHD3 | 2021 | - | - | 0/- | 0/- | 27.6/- | 0/- | 0/- | 0/- | 0/- | 0/- | 0/- | 0/- | 65.0/- | 0/- | 0/- | 2 | 93.10 | - | - |
| BJCP5 | 2021 | - | - | - | - | - | - | - | - | - | - | - | - | - | - | - | - | - | > 13000 | - |
| BJPG3 | 2021 | - | - | 0/- | 0/- | 100/- | 0/- | 0/- | 0/- | 0/- | 4.0/- | 0/- | 14.0/- | 0/- | 0/- | 0/- | 2 | 100 | - | - |
| BJTZ2 | 2021 | - | - | 0/- | 0/- | 80.0/- | 0/- | 0/- | 0/- | 0/- | 12.5/- | 0/- | 0/- | 0/- | 0/- | 0/- | 2 | 87.5 | - | - |
| NMHH2 | 2021 | -/0 | -/0 | 0/0 | 0/0 | 0/5.5 | 0/0 | 0/0 | 0/0 | 0/0 | 0/0 | 0/0 | 0/0 | 0/0 | 0/0 | 0/0 | 1 | 0 | 2.19 | 0 |
| NXGY2 | 2021 | - | - | 0/- | 0/- | 0/- | 0/- | 0/- | 0/- | 0/- | 0/- | 0/- | 0/- | 0/- | 0/- | 0/- | 0 | 0 | 0.61 | 0 |
| SCCD2 | 2021 | - | - | 0/- | 0/- | 0/- | 0/- | 0/- | 0/- | 0/- | 0/- | 0/- | 0/- | 0/- | 0/- | 0/- | 0 | 0 | - | - |
| SDQZ | 2021 | -/0 | -/0 | 0/0 | 41.2/14.2 | 0/11.8 | 0/0 | 5.0/7.9 | 0/0 | 0/0 | 0/0 | 0/0 | 0/0 | 0/0 | 0/0 | 0/0 | 3 | 75 | 6832.26 | 50 |
| SDSG3 | 2021 | -/0 | -/50.75 | 0/0 | 0/2.5 | 3.2/6.6 | 0/0 | 0/1.7 | 0/0 | 0/0 | 0/4.3 | 0/0 | 0/0 | 0/0 | 0/0 | 0/0 | 5 | 3.23 | 1606.15 | 31.7 |
| SDSG4 | 2021 | - | - | 0/- | 61.1/- | 2.8/- | 0/- | 6.3/- | 0/- | 0/- | 1.6/- | 0/- | 0/- | 0/- | 0/- | 0/- | 4 | 84.21 | 36.84% | 76.1 |
| SDSG5 | 2021 | - | - | 0/- | 1.8/- | 1.8/- | 0/- | 31.7/- | 0/- | 0/- | 0/- | 0/- | 0/- | 0/- | 0/- | 0/- | 3 | 50 | 3103.07 | 33.9 |
| SDSG6 | 2021 | - | - | 0/- | 0/- | 1.9/- | 0/- | 88.0/- | 0/- | 0/- | 0/- | 0/- | 0/- | 0/- | 0/- | 0/- | 2 | 100 | 23612.38 | 55.4 |
| YNKM2 | 2021 | - | - | 0/- | 5.0/- | 5.0/- | 0/- | 0/- | 0/- | 0/- | 57.81/- | 0/- | 0/- | 6.25/- | 0/- | 0/- | 4 | 77.42 | 2639.36 | 47.4 |
| BJMY | 2024 | - | - | - | - | - | - | - | - | - | - | - | - | - | - | - | - | - | 5542.88 | - |
| SDSG7 | 2024 | - | - | - | - | - | - | - | - | - | - | - | - | - | - | - | - | - | 6106.12 | - |
| BJHD4 | 2024 | - | - | 0/- | 0/- | 15.0/- | 0/- | 0/- | 0/- | 0/- | 0/- | 0/- | 0/- | 0/- | 0/- | 0/- | 1 | 15.00 | 3.08 | 4.17 |
| BJDX5 | 2024 | - | - | 0/- | 0/- | 27.08/- | 0/- | 0/- | 0/- | 0/- | 6.25/- | 0/- | 6.25/- | 0/- | 0/- | 0/- | 3 | 58.33 | 2036.90 | 39.22 |
| BJDX6 | 2024 | -/0 | -/0 | 0/0 | 0/0 | 64.58/89.8 | 0/0 | 0/0.38 | 0/0 | 0/0 | 0/0 | 27.08/0 | 56.25/11.02 | 0/0 | 0/0 | 0/0 | 4 | 100.00 | 7914.83 | 77.97 |
| BJDX7 | 2024 | - | - | 0/- | 0/- | 85.71/- | 0/- | 0/- | 0/- | 0/- | 9.09/- | 0/- | 9.09/- | 0/- | 0/- | 0/- | 2 | 100.00 | 3922.24 | 64.86 |
| GXNN | 2024 | -/51.26 | -/0 | 0/0 | 0/0 | 39.58/31.02 | 0/0 | 0/2.09 | 0/0 | 0/0 | 0/0 | 0/0 | 16.7/18.06 | 0/0 | 0/0 | 0/0 | 4 | 66.67 | 1498.59 | 27.66 |
| GZGY | 2024 | - | - | 0/- | 0/- | 70.83/- | 0/- | 0/- | 0/- | 0/- | 2.08/- | 2.08/- | 47.92/- | 0/- | 0/- | 0/- | 4 | 91.67 | - | - |
| HNCS2 | 2024 | -/0 | -/0 | 0/0 | 0/0 | 35.42/23.53 | 0/0 | 0/0 | 0/0 | 0/0 | 0/0 | 0/0 | 5.88/1.91 | 0/0 | 0/0 | 0/0 | 2 | 64.71 | 30.13 | 1.887 |
| JXYC | 2024 | - | - | 0/- | 0/- | 10.42/- | 0/- | 0/- | 0/- | 0/- | 0/- | 0/- | 8.33/- | 0/- | 0/- | 0/- | 2 | 20.83 | - | - |
| LNDD | 2024 | -/0 | -/0 | 0/0 | 0/0 | 100/99.01 | 0/0 | 0/0 | 0/0 | 0/0 | 56.82/38.38 | 0/0 | 15.91/13.24 | 0/0 | 0/0 | 0/0 | 3 | 100.00 | 3364.80 | 60.94 |
| QHHD | 2024 | -/0 | -/0 | 0/0 | 2.08/0 | 75/71.16 | 0/0 | 0/0 | 0/0 | 0/0 | 34.78/33.76 | 0/0 | 0/0 | 0/0 | 0/0 | 0/0 | 3 | 100.00 | 4590.82 | 55.56 |
| SCCD3 | 2024 | - | - | 0/- | 0/- | 60.42/- | 0/- | 0/- | 0/- | 0/- | 6.52/- | 0/- | 13.04/- | 0/- | 0/- | 0/- | 3 | 82.61 | - | - |
| SDSG8 | 2024 | - | - | 27.08/- | 0/- | 0/- | 0/- | 0/- | 10.87/- | 0/- | 0/- | 0/- | 0/- | 0/- | 0/- | 0/- | 1 | 65.22 | - | - |
| SDWF | 2024 | - | - | 0/- | 2.08/- | 10.42/- | 0/- | 72.92/- | 0/- | 0/- | 2.08/- | 0/- | 0/- | 0/- | 0/- | 0/- | 4 | 100.00 | - | - |
| YNKM3 | 2024 | - | - | 0/- | 93.75/- | 0/- | 0/- | 0/- | 0/- | 0/- | 0/- | 0/- | 0/- | 0/- | 0/- | 43.75/- | 1 | 100.00 | - | - |
| YNKM4 | 2024 | - | - | 0/- | 0/- | 0/- | 0/- | 0/- | 0/- | 0/- | 100/- | 0/- | 0/- | 0/- | 0/- | 0/- | 1 | 100.00 | - | - |
| YNYX | 2024 | -/0 | -/0 | 0/0 | 66.5/60.87 | 0/1.97 | 0/0 | 0/0 | 0/0 | 0/0 | 28.26/24 | 0/0 | 2.17/0 | 0/0 | 6.52/11.56 | 10.87/0 | 3 | 100.00 | 2956.60 | 55.56 |
| ZJHZ1 | 2024 | -/18.38 | -/0 | 0/0 | 0/0 | 83.33/78.21 | 0/0 | 0/0.68 | 0/0 | 0/0 | 10.42/9.72 | 0/0 | 20.83/18.65 | 0/0 | 0/0 | 0/0 | 5 | 100.00 | 6917.85 | 73.33 |
| ZJHZ2 | 2024 | -/12.43 | -/0 | 0/0 | 0/0 | 79.17/76.89 | 0/0 | 0/0 | 0/0 | 0/0 | 0/0 | 0/0 | 2.08/2.0 | 0/0 | 0/0 | 0/0 | 3 | 95.83 | 1884.11 | 29.79 |
| LabS | 2018 | -/0 | -/0 | -/0 | -/0 | -/0 | -/0 | -/0 | -/0 | -/0 | -/0 | -/0 | -/0 | -/0 | -/0 | -/0 | 0 | - | 1.21 | 0 |
| LabR | 2018 | -/0 | -/0 | 0/0 | 0/0 | 100/100 | 0/0 | 0/0 | 0/0 | 0/0 | 100/100 | 0/0 | 0/0 | 0/0 | 0/0 | 0/0 | 2 | 100 | 54335.80 | 84 |
