# Supplementary material for: Recurrent mutations drive the rapid evolution of pesticide resistance in the two-spotted spider mite Tetranychus urticae
Source: eLife. 2025 Aug 11;14:RP106288. doi: 10.7554/eLife.106288 (PMC12339004; doi:10.7554/eLife.106288)
Supplement: Supplementary file 3. [file elife-106288-supp3.docx]

**Supplementary File 3. Laboratory selection and cross-resistance study of *Tetranychus* *urticae***

| Acaricide | Population | Regression (y=) | LC_50_ (95% CI) / (mg/L) | r | RR |
| --- | --- | --- | --- | --- | --- |
| Cyetpyrafen | F1 | 1.96+2.39x | 18.58 (14.85~22.23) | 0.97 | 1.0 |
|  | F32, 16 times of selection, 2019.4.2 | 1.47+1.91x | 70.94 (58.36~92.35) | 0.99 | 3.8 |
|  | F54, 28 times of selection, 2019.9.19 | 1.7252x+0.1483 | 649.12 (513.35~895.95） | 0.98 | 34.9 |
|  | F60, 30 times of selection, 2019.11.27 | 0.8799x+2.1227 | 1862.40 (1112.58~5657.60) | 0.96 | 100.2 |
|  | F62, 31 times of selection, 2019.12.25 | 2.0722x-2.6048 | 4675.77 (3477.70~10856.77） | 0.99 | 251.7 |
|  | F66, 33 times of selection, 2020.4 LabR | 2.2271 x -5.5457 | 54335.8 | 0.88 | 2924.4 |
|  | Unselected for 66 generations, LabS | 3.67 x+4.69 | 1.21 (0.29~1.89） | 0.94 | - |
| Cyenopyrafen | LabS | 2.43x+3.60 | 3.76 (3.09~4.99） | 0.94 | 1.0 |
|  | LabR | 1.05x-0.16 | 79917.51 (32597.48~7279096.99) | 0.78 | 21227.0 |
| Cyflumetofen | LabS | 3.25 x+2.36 | 6.47 (3.58~8.86) | 0.95 | 1.0 |
|  | LabR | 0.62x+1.52 | 452003.19 | 0.97 | 69902.4 |
| *Pyridaben* | LabS | 1.47x-0.25 | 3763.36 (3013.428~5446.99） | 0.97 | 1.0 |
|  | LabR | 1.01x-0.94 | 10351.97 (5899.42~71346.098） | 0.94 | 3.2 |
| Bifenazate | LabS | 2.34x+2.67 | 9.87 (8.3894~11.4358） | 0.99 | 1.0 |
|  | LabR | 2.20x+2.28 | 17.39(14.62~21.35） | 0.98 | 1.8 |
