# Supplementary material for: Recurrent mutations drive the rapid evolution of pesticide resistance in the two-spotted spider mite Tetranychus urticae
Source: eLife. 2025 Aug 11;14:RP106288. doi: 10.7554/eLife.106288 (PMC12339004; doi:10.7554/eLife.106288)
Supplement: Supplementary file 4. [file elife-106288-supp4.docx]

**Supplementary File 4. Sequencing information and genetic diversity of 22 populations of the two-spotted spider mite *Tetranychus* *urticae*.** N, number of individuals for pooled sequencing. π, nucleotide diversity.

| Code | Date | Resistance status | N | Raw_reads | Mapped_reads | Mean_depth | No. of SNP | π | Tajima’ D |
| --- | --- | --- | --- | --- | --- | --- | --- | --- | --- |
| SXYQ | 2017.03.14 | Unkonwn | 300 | 226,518,938 | 165,721,999 | 277.66X | 3,408,816 | 0.00638±0.00010 | 0.4210±0.0219 |
| AHHN | 2017.03.16 | Unkonwn | 50 | 174,220,938 | 168,955,686 | 208.99X | 2,619,282 | 0.00644±0.00010 | 0.3001±0.0228 |
| SDRZ | 2017.03.22 | Unkonwn | 300 | 223,695,602 | 138,326,975 | 230.89X | 2,870,693 | 0.00761±0.00011 | 0.2411±0.0150 |
| SCCD1 | 2017.03.30 | Unkonwn | 200 | 199,016,876 | 166,311,650 | 278.69X | 4,628,586 | 0.00811±0.00010 | -0.0305±0.0158 |
| HNHK | 2017.03.30 | Unkonwn | 300 | 209,078,536 | 133,365,977 | 223.88X | 2,196,442 | 0.00523±0.00010 | 0.2197±0.0232 |
| SHPD | 2017.04.13 | Unkonwn | 300 | 254,783,024 | 176,667,923 | 296.06X | 3,073,153 | 0.00650±0.00010 | 0.3568±0.0198 |
| HNCS1 | 2017.04.02 | Unkonwn | 300 | 209,671,400 | 151,838,290 | 254.52X | 4,149,620 | 0.00726±0.00010 | 0.1330±0.0190 |
| JXNC | 2017.05.08 | Unkonwn | 300 | 145,020,244 | 99,536,930 | 167.41X | 2,225,209 | 0.00552±0.00010 | 0.1681±0.0218 |
| NMHH2 | 2021.06.13 | Susceptible | 300 | 220,519,748 | 149,102,857 | 249.51X | 2,835,462 | 0.00758±0.00011 | 0.1967±0.0151 |
| BJCP4 | 2021.04.27 | Resistant | 230 | 223,631,126 | 139,731,804 | 234.65X | 2,898,958 | 0.00745±0.00011 | 0.1201±0.0155 |
| SDQZ | 2021.06.13 | Resistant | 200 | 188,858,914 | 119,965,047 | 197.17X | 3,231,043 | 0.00743±0.00011 | 0.2025±0.0180 |
| SDSG3 | 2021.04.18 | Resistant | 250 | 188,571,660 | 125,730,235 | 209.41X | 3,109,064 | 0.00687±0.00011 | 0.1560±0.0202 |
| HNCS2 | 2024.01.29 | Susceptible | 300 | 222,928,412 | 151,838,290 | 254.52X | 3,046,516 | 0.00741±0.00010 | 0.1015±0.0151 |
| BJDX6 | 2024.03.15 | Resistant | 300 | 283,068,416 | 191,429,676 | 322.60X | 3,056,055 | 0.00731±0.00010 | 0.0375±0.0153 |
| GXNN | 2024.03.31 | Resistant | 100 | 342,901,124 | 157,079,428 | 264.42X | 3,458,964 | 0.00793±0.00011 | 0.0967±0.0156 |
| LNDD | 2024.03.10 | Resistant | 250 | 306,161,484 | 176,939,299 | 296.53X | 2,723,643 | 0.00722±0.00010 | 0.2689±0.0161 |
| QHHD | 2024.02.29 | Resistant | 50 | 240,992,080 | 135,847,928 | 228.76X | 2,961,593 | 0.00767±0.00011 | 0.1533±0.0168 |
| YNYX | 2024.03.05 | Resistant | 150 | 233,843,470 | 140,711,946 | 236.87X | 2,510,601 | 0.00665±0.00010 | 0.3204±0.0192 |
| ZJHZ1 | 2024.03.15 | Resistant | 400 | 257,063,980 | 146,899,909 | 247.31X | 3,105,779 | 0.00737±0.00010 | 0.0960±0.0154 |
| ZJHZ2 | 2024.03.15 | Resistant | 400 | 210,338,514 | 132,308,752 | 222.79X | 3,306,753 | 0.00747±0.00010 | 0.0585±0.0156 |
| LabS | 2018.04 | Susceptible | 200 | 136,765,686 | 99,182,723 | 165.93X | 2,868,547 | 0.00701±0.00010 | 0.2366±0.0175 |
| LabR | 2020.05 | Resistant | 200 | 130,487,654 | 96,193,157 | 160.86X | 2,958,783 | 0.00680±0.00010 | 0.1220±0.0180 |
| Total number |  |  | 5680 |  |  |  |  |  |  |
