# Supplementary material for: Recurrent mutations drive the rapid evolution of pesticide resistance in the two-spotted spider mite Tetranychus urticae
Source: eLife. 2025 Aug 11;14:RP106288. doi: 10.7554/eLife.106288 (PMC12339004; doi:10.7554/eLife.106288)
Supplement: Supplementary file 5. [file elife-106288-supp5.docx]

**Supplementary File 5. Distribution of haplotypes carrying amino acid mutations in populations.** Population codes in red indicate the resistant population. Haplotype codes in blue indicates the putative ancestral haplotype, while those in red indicate those carrying the potential resistant mutations.

| Population | *sdhB* | | | | | | | | | | |  | *sdhD* | | | | | | | | | | | | | | | | | | | | | | | |
| --- | --- | --- | --- | --- | --- | --- | --- | --- | --- | --- | --- | --- | --- | --- | --- | --- | --- | --- | --- | --- | --- | --- | --- | --- | --- | --- | --- | --- | --- | --- | --- | --- | --- | --- | --- | --- |
|  | H1 | H2 | H3 | H4 | **H5** | **H6** | H7 | **H8** | H9 | H10 | **H11** |  | H1 | H2 | H3 | H4 | **H5** | **H6** | **H7** | **H8** | H9 | **H10** | H11 | H12 | **H13** | H14 | H15 | **H16** | **H17** | H18 | H19 | **H20** | **H21** | **H22** | H23 | H24 |
| BJCP1 | 13 | 3 | 4 | 2 |  |  |  |  |  |  |  |  | 5 | 6 | 1 | 1 |  |  |  |  |  |  |  |  |  |  |  |  |  |  |  |  |  |  |  |  |
| BJPG1 | 3 |  |  | 9 |  |  |  |  |  |  |  |  | 1 | 1 | 1 |  |  |  |  |  |  |  | 3 | 1 |  |  |  |  |  |  |  |  |  |  |  |  |
| BJCP2 | 7 |  | 4 | 16 |  |  |  |  |  |  |  |  |  | 3 | 13 |  |  |  |  |  |  |  |  |  |  |  |  |  |  |  |  |  |  |  |  |  |
| BJHD1 | 11 |  | 1 | 4 |  |  |  |  |  |  |  |  |  | 19 |  |  |  |  |  |  |  |  |  |  |  |  |  |  |  |  |  |  |  |  |  |  |
| BJPG2 | 10 |  |  | 9 |  |  | 8 |  |  |  |  |  | 6 | 10 | 1 |  |  |  |  |  |  |  | 3 |  |  |  |  |  |  |  |  |  |  |  |  |  |
| BJTZ1 | 16 |  | 3 | 8 |  |  |  |  |  |  |  |  | 12 | 16 | 1 |  |  |  |  |  |  |  | 5 |  |  |  |  |  |  |  |  |  |  |  |  |  |
| HNCS1 |  |  | 1 | 26 |  |  |  |  |  |  |  |  | 4 | 7 |  |  |  |  |  |  |  |  | 15 |  |  | 3 |  |  |  |  |  |  |  |  |  |  |
| HNHK | 4 |  |  | 16 |  |  |  |  |  |  |  |  | 4 | 14 |  |  |  |  |  |  |  |  | 4 | 1 |  |  |  |  |  |  |  |  |  |  |  |  |
| SCCD1 | 10 |  | 1 | 8 |  |  | 1 |  |  |  |  |  | 7 | 1 | 4 |  |  |  |  |  |  |  | 1 |  |  |  |  |  |  |  |  |  |  |  |  |  |
| SDRZ | 10 |  | 2 | 11 |  |  | 3 |  |  |  |  |  | 2 | 13 | 1 |  |  |  |  |  |  |  | 2 |  |  |  |  |  |  |  |  |  |  |  |  |  |
| SDSG1 | 3 |  |  | 1 |  |  |  |  |  |  |  |  | 7 |  | 1 |  |  |  |  |  |  |  |  |  |  |  |  |  |  |  |  |  |  |  |  |  |
| SHPD | 1 |  |  | 22 |  |  |  |  |  |  |  |  |  | 5 | 18 |  |  |  |  |  |  |  |  |  |  |  |  |  |  |  |  |  |  |  |  |  |
| BJHD2 | 6 |  |  | 1 |  |  |  |  |  |  |  |  | 1 |  |  |  |  |  |  | **11** |  |  |  |  |  |  |  |  |  |  |  |  |  |  |  |  |
| HNZZ | 7 |  | 4 | 10 |  |  |  |  |  |  |  |  | 2 | 5 | 8 |  |  |  |  |  |  |  | 2 |  |  |  | 1 |  |  |  |  |  |  |  |  |  |
| LNSY | 3 |  |  | 6 |  |  | 1 |  |  |  |  |  | 3 | 6 |  |  |  |  |  |  |  |  |  |  | **1** |  |  |  | **3** |  |  |  |  |  |  |  |
| SDQD | 2 | 10 | 1 | 4 |  |  | 5 |  |  |  |  |  | 1 | 1 | 2 |  |  |  |  |  |  |  | 14 |  |  |  |  |  |  | 1 |  |  |  |  |  |  |
| SDSG2 | 12 |  |  | 4 |  |  |  |  |  |  |  |  | 2 |  |  |  |  |  |  |  |  |  |  |  |  |  |  |  |  |  |  | **10** |  |  |  |  |
| SXAK | 19 |  |  | 2 |  |  |  |  |  |  |  |  |  | 20 |  |  |  |  |  |  |  |  | 2 |  |  |  |  |  |  |  |  |  |  |  |  |  |
| YNKM1 | 6 | 1 | 1 | 12 |  |  |  |  |  |  |  |  | 1 | 2 | 7 |  |  |  |  |  |  |  | 6 |  |  |  |  |  |  |  |  |  |  |  |  |  |
| **BJCP4** | 2 |  | 1 | 5 | **6** |  |  |  |  |  |  |  |  | 1 | 1 |  | **20** |  |  |  |  |  |  |  |  |  |  |  |  |  |  |  |  |  |  |  |
| BJHD3 | 2 |  |  | 11 |  | **2** |  |  |  |  |  |  |  |  | 4 |  |  |  |  | **15** |  |  |  |  |  |  |  |  |  |  |  |  |  |  |  |  |
| BJPG3 |  |  |  |  | **2** | **11** |  |  |  |  |  |  | 3 | 8 | 2 |  |  |  |  |  |  | **1** | 2 |  | **4** |  |  |  |  |  |  |  |  |  |  |  |
| BJTZ2 | 1 |  |  |  | **2** | **3** |  |  |  |  |  |  | 5 | 7 | 4 |  |  |  |  |  |  | **1** |  |  |  |  |  |  |  |  |  |  |  |  |  |  |
| NMHH2 | 14 |  | 1 | 1 |  |  |  |  |  |  |  |  |  |  |  |  |  |  |  |  |  |  | 1 |  |  |  |  |  |  |  |  |  |  |  |  |  |
| NXGY2 | 20 |  | 1 | 2 |  |  | 2 |  |  |  |  |  |  | 2 | 7 |  |  | **6** |  |  |  |  |  |  |  |  |  |  |  |  |  |  |  |  |  |  |
| SCCD2 | 4 |  | 2 | 9 |  |  | 15 |  |  |  |  |  |  | 16 |  |  |  |  |  |  |  |  |  |  |  |  |  |  |  |  |  |  |  |  |  |  |
| **SDQZ** | 4 |  | 1 |  |  |  |  | **2** |  |  |  |  | 8 | 2 | 2 |  |  |  |  |  |  |  |  |  |  |  |  |  |  |  | 4 |  |  |  |  |  |
| **SDSG3** | 9 |  | 1 | 20 |  | **1** |  |  |  |  |  |  | 24 |  | 3 |  |  |  |  |  |  |  |  |  |  |  |  |  |  |  |  |  |  |  |  |  |
| SDSG4 |  |  |  | 2 |  |  |  | **7** |  |  |  |  | 7 | 2 | 3 |  |  |  |  |  |  |  | 3 |  |  | 3 |  |  |  |  |  |  | **1** |  |  |  |
| SDSG5 | 20 |  | 1 | 5 |  |  |  |  |  |  |  |  | 8 |  | 1 |  |  |  |  |  |  |  |  |  |  |  |  |  |  |  |  | **6** |  |  |  |  |
| SDSG6 | 3 |  | 1 | 19 |  |  |  |  | 1 |  |  |  |  |  |  |  |  |  |  |  |  |  |  |  |  |  |  |  |  |  |  |  | **14** |  |  |  |
| YNKM2 | 8 |  | 5 | 10 |  |  |  |  |  |  |  |  |  |  |  |  | **16** |  |  |  |  |  |  |  |  |  |  |  |  |  |  |  |  | **1** |  |  |
| BJDX5 | 5 |  |  | 1 |  | **1** |  |  |  |  |  |  | 1 | 4 |  |  |  |  |  |  |  |  |  |  |  |  |  |  |  |  |  |  |  |  |  |  |
| **BJDX6** |  |  |  | 2 |  | **9** |  |  |  |  |  |  |  |  |  |  |  | **1** | **1** |  |  |  |  |  |  |  |  |  |  |  |  |  |  |  |  |  |
| BJDX7 |  |  |  | 1 |  | **13** |  |  |  |  |  |  | 1 | 1 |  |  |  |  |  |  |  |  |  |  |  |  |  |  |  |  |  |  |  |  |  |  |
| BJHD4 | 3 |  |  |  |  | **1** |  |  |  |  |  |  |  |  | 6 |  |  |  |  |  | 1 |  |  |  |  |  |  |  |  |  |  |  |  |  |  |  |
| **GXNN** | 2 |  |  | 7 | **2** | **4** |  |  |  |  |  |  | 1 |  |  |  |  | **1** |  |  |  |  |  |  |  |  |  |  |  |  |  |  |  |  |  |  |
| GZGY | 2 |  |  |  | **1** | **6** |  |  |  |  |  |  | 1 |  |  |  |  |  |  |  |  |  |  |  |  |  |  |  |  |  |  |  |  |  |  |  |
| **HNCS2** |  |  |  | 6 |  | **2** |  |  |  |  |  |  | 1 | 4 | 1 |  |  |  |  |  |  |  |  |  |  |  |  |  |  |  |  |  |  |  |  |  |
| JXYC | 7 |  |  | 3 |  | **2** | 2 |  |  |  |  |  | 5 | 5 |  |  |  |  |  |  |  |  |  |  |  |  |  | **1** |  |  |  |  |  |  |  |  |
| **LNDD** |  |  |  |  | **10** | **3** |  |  |  |  |  |  |  | 1 |  |  |  |  |  |  |  |  |  |  |  |  |  |  | **3** |  |  |  |  |  |  |  |
| **QHHD** |  | 1 |  |  |  | **12** |  |  |  |  |  |  |  | 1 | 2 |  |  |  |  |  |  |  |  |  |  |  |  |  | **2** |  |  |  |  |  |  |  |
| SCCD3 | 1 |  |  | 2 |  | **10** |  |  |  |  |  |  | 1 | 2 | 1 |  |  |  |  |  |  |  | 1 |  |  |  |  |  |  |  |  |  |  |  |  |  |
| SDSG8 | 12 |  |  |  |  |  |  |  |  | 2 |  |  |  | 5 | 3 |  |  |  |  |  |  |  |  |  |  |  |  |  |  |  |  |  |  |  |  |  |
| SDWF | 9 |  |  |  |  |  |  |  |  |  |  |  |  |  |  |  |  |  |  |  |  |  |  |  |  |  |  |  |  |  |  | **10** |  |  |  |  |
| YNKM3 |  |  |  | 4 |  |  |  | **5** |  |  | **5** |  |  | 1 |  |  | **23** |  |  |  |  |  |  |  |  |  |  |  |  |  |  |  |  |  | 4 |  |
| YNKM4 | 9 |  |  |  |  |  |  |  |  |  |  |  |  |  |  |  |  |  |  |  |  |  |  |  |  |  |  |  |  |  |  |  |  |  |  |  |
| **YNYX** |  |  |  | 2 |  |  |  | **2** |  |  | **1** |  | 6 |  |  |  | **1** |  |  |  |  |  |  |  |  |  |  |  |  |  |  |  |  |  |  |  |
| **ZJHZ1** |  |  |  |  |  | **18** |  |  |  |  |  |  |  |  |  |  | **1** | **1** |  |  |  |  |  |  |  |  |  |  |  |  |  |  |  |  |  |  |
| **ZJHZ2** |  |  |  |  |  | **11** |  |  |  |  |  |  |  | 5 | 1 |  |  |  |  |  |  |  | 3 |  |  |  |  |  |  |  |  |  |  |  |  |  |
| ZJXS1 | 14 |  | 1 | 17 |  |  | 4 |  |  |  |  |  | 2 | 11 | 4 | 1 |  |  |  |  |  |  | 10 |  |  |  |  |  |  |  |  |  |  |  |  | 1 |
| **LabR** |  |  |  |  |  | **24** |  |  |  |  |  |  |  |  |  |  |  |  |  |  |  | **32** |  |  |  |  |  |  |  |  |  |  |  |  |  |  |
