# Supplementary material for: Recurrent mutations drive the rapid evolution of pesticide resistance in the two-spotted spider mite Tetranychus urticae
Source: eLife. 2025 Aug 11;14:RP106288. doi: 10.7554/eLife.106288 (PMC12339004; doi:10.7554/eLife.106288)
Supplement: Supplementary file 6. [file elife-106288-supp6.docx]

**Supplementary File 6. Correlation between eight mutations on SDH genes and level of resistance (survival percentages under 1000 mg/L)**

| **Mutations** | **Regression (y =)** | **Coefficient (R2)** |
| --- | --- | --- |
| sdhB_I260T | 0.526x + 0.358 | 0.0927 |
| sdhB_I260V | -0.00222x + 0.4 | 0.0031 |
| sdhD_R119C | 0.496x + 0.318 | 0.198 |
| sdhD_R119L | 0.808x + 0.353 | 0.0981 |
| sdhD_R119G | 1.47x + 0.381 | 0.0647 |
| sdhD_R119H | 0.769x + 0.394 | 0.00107 |
| sdhD_R119P | -0.474x + 0.413 | 0.0669 |
| sdhD_D116G | 0.4967x + 0.2937 | 0.1605 |
| Predominant resistant allele | 0.757x + 0.0401 | 0.703 |
| Individuals with at least one resistant allele | 0.649x - 0.0088 | 0.747 |
| Individuals with at least one homozygous resistant genotype | 0.702x + 0.132 | 0.606 |
